# A Supervised Pattern Analysis of the Length of Stay for Hip Replacement Admissions

**DOI:** 10.3390/healthcare7020058

**Published:** 2019-04-06

**Authors:** Dimitrios Zikos, Ashara Shrestha, Taylor Colotti, Leonidas Fegaras

**Affiliations:** 1 School of Health Sciences, Central Michigan University, Mt. Pleasant, MI 48859, USA; colot1te@cmich.edu; 2 Computer Science Department, University of Texas at Arlington, Arlington, TX 76019, USA; aashara.shrestha@mavs.uta.edu (A.S.); fegaras@cse.uta.edu (L.F.)

**Keywords:** hip replacement, length of stay, hospital, Medicare, pattern analysis

## Abstract

Hip replacement is the most common surgical procedure among Medicare patients in the US and worldwide. The hospital length of stay (LOS) for hip replacement admissions is therefore important to be controlled, contributing to savings for hospitals. This study combined medical claims and hospital structure and service data to examine LOS fluctuations and trends, and admission distribution patterns, during weekdays, for hip replacement cases. The study furthermore examined associations of these patterns with the LOS performance. Most hospitals were found to admit hip replacement cases at the start of the week (Monday through Wednesday). There is an upward LOS trend as we approach late weekday admissions. Multiple linear regression analysis showed that LOS weekday inconsistencies, a large proportion of hip replacement admissions on Thursday and Friday, the government ownership status, the bed size, and the critical access status are associated with an increased LOS. On the other hand, the rate of hip replacement admissions over total ones, and the hospital being accredited, are associated with a lower LOS. Findings stress out the need for hospitals to maintain an effective and balanced distribution of hip replacement admissions, evenly during the week, and the need for standardized case management, to avoid practice variability and, therefore, LOS fluctuations for their hip replacement cases.

## 1. Introduction

In the United States, length of stay (LOS) in hospitals is used as an efficiency indicator [1], putting enormous pressure on staff and management to keep LOS to a minimum [2]. Typically, a shorter stay reduces the cost per discharge. Since hospitals account for about 40% of health care costs in America [3], shortening LOS seems to be a popular strategy to cut costs. However, cases admitted over a weekend, the eve of a holiday, in the afternoons, and after office hours, do have an increased LOS [4]. Of the 39.5 million community hospital stays in 2007, 7.7 million stays or about 19 percent began on a weekend [5]. Previous research found that hospital admission on weekends was associated with increased mortality, greater LOS, and delay in performance of procedures for certain conditions [5,6]. Ryan et al. found that patients admitted on weekends tend to experience delays in receiving major procedures. On the day of admission, weekend-admitted patients received 36% of the major procedures that they would receive during their stays, compared to 65% for patients admitted on weekdays [5]. Hospitals that are faced with economic constraints and problems of employee satisfaction usually have reduced staffing and availability of services on the weekends [7]. To address this problem, as it is known that increased weekend continuity of care is associated with reduced LOS, improvement in weekend cross-coverage and patient handoffs may be useful to improve clinical outcomes [8].

Currently, hospitals make decisions on the admission date based on a number of factors, including operating rooms available, types of equipment purchased, the quality and mix of surgical assistants and nursing staff, and the organization of the operating room schedule itself [9]. Physicians also play a role in this. Surgeons may choose an admission date based on their choice of a given technique for a particular procedure based on prior surgeries performed, or they may be influenced by the hospital’s quality assurance committee. According to Shortell [9], hospital decision making follows a certain two-tiered typology: (1) The degree of agreement or certainty among key parties as to their preferences for specific outcomes and (2) the degree of confidence or certainty in the cause–effect relationships involved. Following this typology, regarding surgical procedures, the specific outcomes and desired results include a successful procedure without complications and a low LOS. Hospitals seem to be aware of the increase in LOS for patients admitted on the weekends, as medical claims data (CMS) show decreased numbers in elective procedure admissions as the weekend approaches, due to the increased probability for these admissions to have to go through the weekend. 

For the purpose of claims data, there are three different types of hospital admissions: Emergency, urgent, and elective. An emergency hospital admission must be conducted immediately because of a life-threatening condition [10], while an urgent admission refers to a condition that needs prompt attention, or else it may worsen. Elective procedures, on the other hand, refer to those surgical procedures that may or may not be planned in advance, rather than one that is done in an emergency situation [11]. Elective procedures may lead to a better quality of life but are not life-threatening situations [10]. Research shows that there is a higher risk of mortality for patients who have elective procedures carried out later in the week and during the weekend, rather than at the beginning of the week [12]. In addition, development of hospital-acquired infections (HAI), specifically surgical site infections (SSIs), after elective surgical procedures is very common, costing the US at least $3.5 billion per year and increasing the LOS by an average of 9.7 days [13]. In the United States, over 1 million hip and knee replacements are performed each year [14], which constitute elective surgical procedures. Due to the aging of the baby boomer generation, these annual volumes are projected to increase dramatically in the future, making joint replacements the most common elective surgical procedure [14].

Medicare is the federal health insurance program in the United States, created in 1965 for people ages 65 and over, regardless of income, medical history, or health status. Currently, over 59 million people are enrolled in Medicare coverage [15], accounting for about 15% of the United States population. Medicare spending accounts for almost 20% of the total national health spending each year, equating to $672.1 billion [16]. The prospective payment system for Medicare patients is based on diagnosis-related groups (DRGs). The use of DRGs standardizes prospective payment to hospitals and encourages cost containment initiatives. In general, a DRG payment covers all charges associated with an inpatient stay from the time of admission to discharge. DRGs categorize patients with respect to diagnosis, treatment, and length of hospital stay. The DRG is dependent upon the principal diagnosis, secondary diagnoses, surgical procedures performed, comorbidities and complications, patient’s age and sex, and discharge status [17]. Hospitals are paid based on the DRG assigned, otherwise known as the “fee-for-service” system. The Medicare fee-for-service system has provided an incentive for shortening length of stay without penalty for potential unfavorable later outcomes, such as increased readmission or mortality rates [18].

For the purpose of this study, we focused on the major joint replacement surgeries of the lower extremities without major complications (DRG = 470) among Medicare patients. We refer to these admissions, in this paper, as hip replacement admissions. We examined, at a hospital level:

(1) Weekday patterns of the LOS for elective hip replacement admissions during the course of the week, and whether the LOS is higher for admissions that take place at the end of the week and lower for early week admissions;
(2) For hip replacement admissions, the association between (a) the hospital LOS and (b) the distribution of admissions during weekdays (including overconcentration) and the LOS weekday fluctuations; control for the proportion of hip replacement cases per hospital, and other hospital characteristics such as hospital size, teaching, ownership status, and offered services;
(3) LOS differences (comparison statistics) among different ownership, accreditation status, teaching status, and hospital services availability indicators.

## 2. Materials and Methods

### 2.1. Data Sources

This research used two data sources. The first one is an inpatient de-identified dataset (LDS) with every Medicare admission in the US for the year 2016. This dataset was purchased by the Centers of Medicare and Medicaid Services (CMS). This dataset includes ~10 million hospital admissions. The second data source was purchased by the American Hospital Association (AHA). These data provide information about staffing and hospital services for every hospital in the US. Both files include the Medicare ID, which was used as a common key to combine the two files. Before the data merging, we aggregated the CMS data at a provider level. For each provider, we later on calculated a number of LOS and admission distribution indices. Non-elective hip replacement admissions were removed, and after the data aggregation at a hospital level, we furthermore removed hospitals with less than 10 elective hip replacement (DRG = 470) cases. This left a total of 2805 hospitals. The analysis was conducted at the hospital level. Table 1 shows the attributes that were used in this study.

### 2.2. Calculation of Length of Stay Indices and Admission Distribution Indices

Since the study objective was to investigate LOS trends, LOS stability and admission overconcentration, and their effect on the hospital LOS performance, we calculated, for each hospital, a number of indicators and added these new data to the analysis file.

‘LOS Weekday Trend Index’: This indicator describes, for each hospital, the length of stay trend as we move from Monday to Friday. It was calculated for each hospital as the Pearson correlation between the five weekdays, ordered, and the distribution {1, 2, 3, 4, 5}. The index takes values from −1 through to 1. A positive index indicated an upward trend of the length of stay as we move from Monday through to the end of the week. The closest to 1, the strongest the LOS increase trend is.

$$\mathrm{LOS}\;\mathrm{Weekday}\;\mathrm{Trend}\;\mathrm{Index}\;=\frac{5*\sum \left(k*d\right)-\sum \left(k\right)*\sum \left(d\right)}{\sqrt{\left[5*\sum (k^{2})-{\left(\sum k\right)}^{2}]*[5*\sum d^{2}-{\left(\sum d\right)}^{2}\right]}}$$

where: *k* = {1, 2, 3, 4, 5} and *d* = {LOS_Mon_, LOS_Tue_, LOS_Wed_, LOS_Thu_, LOS_Fri_}.

Weekday LOS Stability Index: This indicator describes the stability of the length of stay during weekdays for each hospital. For each hospital, we calculated the standard deviation of its mean length of stays for the five weekdays. The index takes values from 0–∞. The lower the index score, the greater the stability of the length of stay along the five-weekday admissions for the hospital.

$$\mathrm{Weekday}\;\mathrm{LOS}\;\mathrm{Stability}\;\mathrm{Index}=\sqrt{\frac{\sum {\left(d-\bar{d}\right)}^{2}}{5}}$$

where: *d* = {LOS_Mon_, LOS_Tue_, LOS_Wed_, LOS_Thu_, LOS_Fri_}.

These two indicators are only moderately dependent (*r* =0.172): It is possible for a hospital to have a high ‘LOS Weekday Trend Index’ score and a low ‘LOS Weekday Stability Index’ score, for instance {Mon: 3.05 days, Tue: 3.10 days, Wed: 3.12 days, Thu: 3.15 days, Fri: 3.20 days}. It is possible, on the other hand, for the opposite to occur, for instance when: {Mon: 3.5 days, Tue: 2.5 days, Wed: 3.5 days, Thu: 2.5 days, Fri: 3.5 days}.

Admission Ratio of DRG = 470 over total: This is the rate of DRG = 470 admissions over total hospital admissions. This variable was calculated to serve as a proxy for the relative hospital experience in serving hip replacement cases.

Admission Overconcentration Indicators: Two ‘Admission Overconcentration Indicators’ were calculated for each hospital: (i) >50% of the hip replacement admissions in a single weekday; (ii) >90% of the DRG = 470 admissions in two weekdays. These indicators are used as admission overconcentration indices. It becomes possible to examine, this way, the association of DRG = 470 overconcentration with the length of stay for hip replacement cases. To calculate the two indicators, we firstly estimated the proportion of hip replacement admissions per weekday (A_Weekday_), for each hospital, and then, we used the following conditionals:

(i) For the ‘>50% of the DRG = 470 admissions in a single weekday’ indicator:
  IF [OR (A_Mon_ > 50%), (A_Tue_ > 50%), (A_Wed_ > 50%), (A_Thu_ > 50%), (A_Fri_ > 50%), (A_Sat_ > 50%), (A_Sun_ > 50%)] = TRUE
  THEN ‘>50% of the DRG = 470 admissions in a single weekday’ = 1
  ELSE ‘>50% of the DRG = 470 admissions in a single weekday’ = 0
(ii) For the ‘>90% of the DRG = 470 admissions in two weekdays’ indicator:
  IF [OR(A_Mon_ + A_Tue_ > 90%), (A_Mon_ + A_Wed_ > 90%), (A_Mon_ + A_Thu_ > 90%), (A_Mon_ + A_Fri_ > 90%), (A_Mon_ + A_Sat_ > 90%), (A_Mon_ + A_Sun_ > 90%), (A_Tue_ + A_Wed_ > 90%), (A_Tue_ + A_Thu_ > 90%), (A_Tue_ + A_Fri_ > 90%), (A_Tue_ + A_Sat_ > 90%), (A_Tue_ + A_Sun_ > 90%), (A_Wed_ + A_Thu_ >90%), (A_Wed_ + A_Fri_ > 90%), (A_Wed_ + A_Sat_ >90%), (A_Wed_ + A_Sun_ > 90%), (A_Thu_ + A_Fri_ > 90%), (A_Thu_ + A_Sat_ > 90%), (A_Thu_ + A_Sun_ >90%), (A_Fru_ + A_Sat_ > 90%), (A_Fri_ + A_Sun_ > 90%), (A_Sat_ + A_Sun_ > 90%)] **= TRUE**
  **THEN** ‘>90% of the DRG = 470 admissions in two weekdays’ = 1
  **ELSE** ‘>90% of the DRG = 470 admissions in two weekdays’ = 0

**Figure 1:**
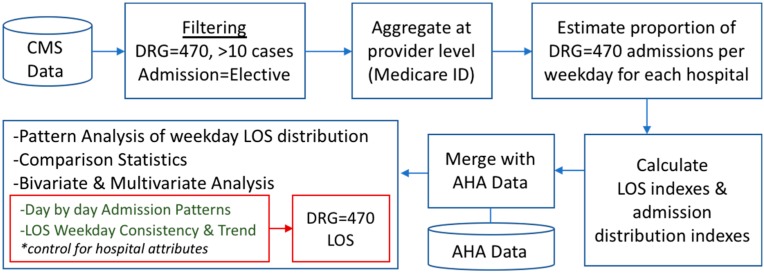
Overview of the research approach.

Weekday DRG = 470 Admission Distribution: A numerical expression of the weekday concentration of admissions. This is the standard deviation of the Monday through Friday admission distribution for each hospital. Figure 1 summarizes the data preparation and analysis approach.

## 3. Results

A total of 2805 hospitals were included in the analysis. These are all hospitals with at least ten inpatient cases of elective hip replacement admissions. Table 2 provides descriptive statistics of the attributes that were examined in this research.

After having calculated the proportion of hip replacement admissions per hospital, for each weekday, we found that most hospitals tend to admit these cases during the first two weekdays (Monday and Tuesday). As Table 3 shows, more than 60% of the total hip replacement admissions take place on Monday and Tuesday.

**Figure 2:**
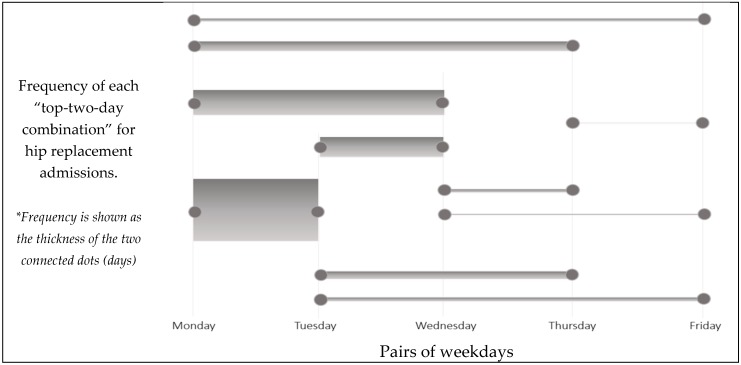
Frequency of top two admission days for elective DRG = 470 cases.

For hip replacement admissions, the mean LOS across all hospitals was found to be 2.62 days (St.Dev. = 0.63 days). The hospital LOS variable was found to follow a normal distribution, according to the Kolmogorov–Smirnov test for normality. An interesting pattern to the hospital-preferred hip replacement admission weekdays was found: Most of the hospitals admitted DRG = 470 cases in consecutive days. We ranked the top and the second most popular admission weekdays for hip replacements, per hospital, and then visualized the count of each of these weekday pairs. Figure 2 shows that the pair of Monday and Tuesday is the most preferred admission day pair for almost half of the hospitals, with the Monday–Wednesday (18.06%) and the Tuesday–Wednesday pairs following (14.18%). This finding may indicate hospital awareness about the significance of early weekday admissions, or the fact that surgeons are available only during specific weekdays, which, in turn, drives hospital admission decisions.

**Figure 3:**
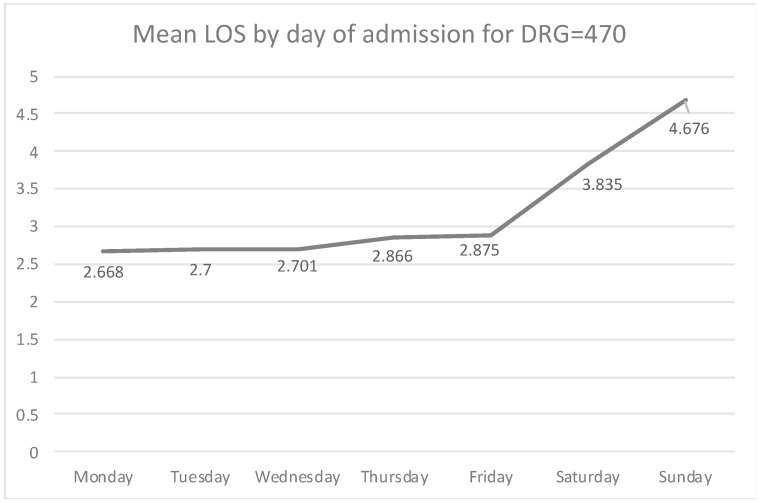
The length of stay (LOS) increases when hip replacement admissions take place at a late weekday.

The mean LOS for elective hip replacements is increasingly higher as we approach admissions that occurred towards the end of the week (Figure 3). Characteristically, the mean LOS for Monday admissions is 2.668 days, slightly increasing on Tuesday (2.700 days) and Wednesday (2.701 days). A more drastic increase is observed later on in the week, with Thursday admissions having a mean stay of 2.866 days, while Friday admissions have a mean LOS of 2.875 days. Weekend admissions are very rare (only 0.28% of elective DRG = 470 admissions happen on weekends), but when this occurs, the LOS for those patients is very high (3.835 for Saturday, and 4.676 days for Sunday).

### 3.1. Correlation Statistics

Length of Stay: There was found to be a statistically significant, moderate negative association between the mean LOS and the proportion of hip replacement admissions over total (Pearson = −0.221, *p* < 0.001). The larger the proportion of hip replacement cases, the lower the mean LOS becomes. In addition, the mean LOS is positively associated with the ‘LOS Weekday Stability Index’ (Pearson = 0.192, *p* < 0.001). The bed count has a weak but statistically significant association with the LOS (Pearson = −0.053, *p* < 0.01). The larger the hospital, the lower the LOS for elective hip replacement admissions becomes.

In addition, the less consistent the LOS during the five weekdays (high ‘LOS Weekday Stability Index’ score), the higher the hospital LOS. This finding holds significant implications in terms of care delivery consistency. A more detailed weekday by weekday investigation revealed that the higher the distribution of admissions on early weekdays (Monday and Wednesday), the lower the mean LOS becomes. The correlation has an opposite direction for Friday admissions: Hospitals which “load up” Fridays and Saturdays with many hip replacement admissions proportionally were found to have a higher LOS for their hip replacement patients (Pearson = 0.061, *p* < 0.001 for Friday, Pearson = 0.106, *p* < 0.001 for Saturday). Table 4 presents the associations between the LOS and the proportion of DRG = 470 admissions for each weekday.

‘LOS Weekday Stability Index’: There is a statistically significant, negative association between the mean ‘LOS Weekday Stability Index’ and the proportion of hip replacement admissions over total (Pearson = −0.118, *p* < 0.001). Hospitals with a higher proportion of hip replacement admissions over total ones may have greater accumulated experience in hip replacement surgeries, and therefore, better overall patient management, resource management, and improved workflows. As explained earlier, the mean LOS was found to be positively associated with the ‘LOS Weekday Stability Index’ (Pearson = 0.192, *p* < 0.001). A less consistent LOS during weekdays (high ‘LOS Weekday Stability Index’ score) is associated with a higher hospital LOS for hip replacement admissions.

‘LOS Weekday Trend Index’: In a similar manner, there was found to be a statistically significant, negative association between the mean ‘LOS Weekday Trend Index’ and the proportion of hip replacement admissions over total ones (Pearson = −0.122, *p* < 0.001). Hospitals with a higher analogy of hip replacement admissions have a lower index score, indicating a lower day-by-day increase to the LOS for their hip replacement cases. In addition, the hospital bed size was positively associated with the ‘LOS Weekday Trend Index’ (Pearson = 0.104, *p* < 0.001). Larger hospitals have a higher index score, which indicates a higher day-by-day increase to the LOS as we approach the weekend.

Distribution of hip replacement admissions during the week: The distribution of hip replacement cases across the five weekdays was found to be positively associated with the LOS (Pearson = 0.164, *p* < 0.001) and the ‘LOS Weekday Stability Index’ (Pearson = 0.129, *p* < 0.001). This means that the more uneven the distribution of hip replacement admissions in the weekdays is, the higher the LOS becomes. Additionally, the ‘LOS Weekday Trend Index’ was found to be weekly but significantly associated with the distribution of admissions (Pearson = −0.049, *p* < 0.05). The distribution index of hip replacement cases across the five weekdays was found to be moderately associated with the rate of hip replacement admissions over total (Pearson = −0.133, *p* < 0.001) and the bed count (Pearson = −0.378, *p* < 0.001): The larger the hospital, the more evenly distributed the hip replacement admissions are, across the five weekdays. Similarly, the higher the proportion of hip-replacement cases over total, the more evenly distributed are the hip replacement admissions across the five weekdays.

### 3.2. Comparison of Hospital Ownership Types in Terms of LOS

Analysis of Variance (ANOVA) was conducted to find if there are differences to the three LOS indices across the three types of organizations (governmental, for-profit, no-profit). The three ownership status types differ in terms of the mean LOS (F = 28.39, *p* < 0.001). Additionally, the three organizational types differ in terms of the ‘Weekday LOS Trend Index’ (F = 7.384, *p* < 0.001). No differences were observed for the ‘Weekday LOS stability index’. Finally, there are significant differences across the three types of ownership for the weekday DRG = 470 admission distribution, that is, how evenly the patient admissions occur during weekdays (F = 27.630, *p* < 0.001). Post-hoc analysis was further conducted (Table 5), with Tukey Honestly Significant Difference (HSD), to compare all pairs of the three hospital ownership status types (government vs. for-profit, government vs. nonprofit, and for-profit vs. nonprofit), for the LOS and the two LOS weekday indices under study. As far as the LOS is concerned, statistically significant differences were observed, between governmental and nonprofit organizations (mean diff = 0.267 days, 95% CI = 0.181–0.354, *p* < 0.001), governmental and for-profit organizations (mean diff = 0.167, 95% CI = 0.065–0.269, *p* < 0.001), and between for-profit and nonprofit organizations (mean diff = 0.100 days, 95% CI = 0.028–0.173, *p* < 0.005). Regarding the ‘LOS Weekday Trend Index’, there is a statistically significant difference between the government and for-profit (mean diff = 0.142 days, 95% CI = 0.036–0.248, *p* < 0.01), and between nonprofit and for-profit hospitals (mean diff. = 0.112 days, 95% CI = 0.038–0.186, *p* < 0.01). No statistically significant differences were observed between the three organization types for the ‘LOS Weekday Stability Index’.

### 3.3. Comparison of the LOS Indices between Hospital Structure Attributes

Hospital characteristics and services availability: Differences were observed to the mean LOS when we compared hospitals based on the existence of hospital characteristics and services. Hospitals that have received accreditation from the Accreditation Council for Graduate Medical Education (ACGMA) were found to have a LOS which is lower by 0.1 days than non-accredited hospitals. By contrast, critical access hospitals have an average LOS which is higher by 0.295 days than noncritical access ones. Table 6 presents all statistically significant differences as observed with independent sample *t*-test analysis.

‘LOS Weekday Trend Index’ Differences: Differences were also observed to the ‘LOS Weekday Trend Index’ when we compared hospitals based on the existence of hospital characteristics and services. Hospitals that have received accreditation from ACGMA, those which offer American Osteopathic Association (AOA) residency, and hospitals that belong to the Council of Teaching Hospitals (AAMC) have a lower ‘LOS Weekday Trend Index’, than non-ACGMA-accredited (by 0.099 days), non-AOA residency hospitals (by 0.090 days), and non-AAMC hospitals (by 0.103 days), respectively. This means that ACGMA, AOA-accredited, and teaching hospitals observe a less drastic day-by-day LOS increase, as we approach the weekend, for hip replacement cases. By contrast, critical access hospitals have a higher ‘LOS Weekday Trend Index’ score (by 0.090 days) than noncritical access ones. Table 7 presents all statistically significant differences. No statistically significant differences were observed for the ‘LOS Weekday Stability Index’.

Length of Stay and DRG = 470 Admission Overconcentration: We compared the average LOS between hospitals with positive and negative hip replacement admission overconcentration indicators. These indicators have been calculated during data preparation, earlier on, to illustrate hospitals that tend to admit the majority of their hip replacement cases on a single or just two weekdays. According to the t-test analysis, the LOS differs between hospitals with a positive and negative overconcentration indicator. Specifically, hospitals with >50% of hip replacement admissions on a single weekday were found to have a higher LOS, by 0.171 days, than hospitals will less than 50% of admissions over concentrated on a single weekday. Similarly, hospitals with >90% of hip replacement admissions on two weekdays have a high hip replacement LOS, by 0.148 days, then hospitals will less than 90% of admissions over concentrated on a just two weekdays (Table 8).

### 3.4. Multivariate Analysis of LOS Predictors for Hip Replacement Admissions

We furthermore conducted multiple linear regression analysis to examine the effect of the LOS stability (*LOS Weekday Stability Index*), LOS weekday trends (*LOS Weekday Trend Index*), and hip replacement admission distribution, on the hospital LOS, for hip replacement admissions. The independent variables that were inserted to the model are a series of hospital structure characteristics and process attributes. Structure characteristics include the hospital size (number of beds), the ownership status, hospital accreditation indicators, teaching status, and a number of service availability indicators, such as the availability of emergency department, cardiology, and rehabilitation services.

Process attributes that were inserted to the model include the ‘DRG = 470 Admission Ratio over total admissions’, the ‘LOS Weekday Stability Index’, the ‘LOS Weekday Trend Index’, and finally, the distribution of DRG = 470 admissions for each day of the week, and the two DRG = 470 overconcentration indicators: *‘>50% of DRG = 470 admissions in a single day’* and *‘>90% of DRG = 470 admissions in two days’*. Finally, the US state variables were also included in the model to examine the State Fixed Effect (FE) on the LOS for DRG = 470 admissions. The model was found to explain 26.6% of the total LOS variance (*R^2^* = 0.266), and the standard error of the estimate was equal to 0.552 days. ANOVA analysis was found to be significant (F = 9.078, *p* < 0.001). Collinearity statistics were conducted, and no multicollinearity was detected among the independent variables of the model. The ‘LOS Stability Index’ was found to have a significant association with the mean LOS (b = 0.134, *p* < 0.001). 

The proportions of Thursday (b = 0.005, *p* < 0.001), Friday (b = 0.006, *p* < 0.001), and weekend DRG = 470 admissions were all found to be positively associated with the LOS (Table 9). The ‘DRG = 470 Admission Ratio over total admissions’ was found to be negatively associated with the LOS (b = −1.926, *p* < 0.001). As far as hospital structure attributes are concerned, the ownership status of a hospital being governmental (b = 0.168, *p* < 0.001) and for-profit (b = 0.318, *p* < 0.001) was found to be positively associated with the LOS. The hospital size (total beds) was found to have a statistically significant association with the LOS (b = 0.001, *p* < 0.01). A negative, marginally statistically significant association was observed between the LOS and the Joint Commission Accreditation Status of the hospital (b = −0.077, *p* = 0.05). Finally, the availability of some hospital services was associated with the LOS: The availability of an emergency department (b = −0.302, *p* < 0.005) and the adult cardiology services (b = −0.083, *p* < 0.05) were found to have a negative association with the LOS. On the other hand, the status of the hospital being a critical access one is positively associated with the LOS in a statistically significant way (b = 0.200, *p* < 0.001). ‘Critical Access Hospital’ is a federal program that offers small hospitals in underserved rural areas that have no access to emergency care.

## 4. Discussion

According to results, hip replacement admissions that occur towards the end of the week have an increased LOS. Starting with a 2.67-day LOS for Monday admissions, the LOS increases gradually for admissions that occur on a later weekday. In addition, bivariate associations between the LOS and weekday admission distribution are negative for Monday admissions and positive for Thursday and Friday admissions. This suggests that hospitals with a high proportion of hip replacement admissions on Monday keep those patients fewer days on average, while hospitals with a high proportion of hip replacement admissions on Thursday and Friday have, on average, a higher LOS. We also found that the majority of hospitals tend to admit hip replacement patients at the beginning of the week. It appears that hospitals are, to some degree, aware that elective hip replacement admissions need to take place at the start of the week. It is unknown, though, whether this is due to hospitals being aware of positive implications of early weekday admissions, or for any other reasons, such as for instance, surgeon availability. In addition, according to results, most hospitals concentrate their admissions on Monday, and especially on early consecutive weekdays (Monday and Tuesday).

The hospital LOS for hip replacement admissions was found to be positively associated with the ‘LOS Weekday Stability Index’: Hospitals that maintain a consistent LOS across the weekdays tend to keep, on average, their hip replacement patients for fewer days. Additionally, hospitals that distribute the hip replacement admissions unevenly during the five weekdays keep their hip replacement patients longer, on average. Hospitals with a higher proportion of hip replacement admissions in total were found to achieve a lower LOS than hospitals with proportionally fewer hip replacement admissions. Further, those hospitals with proportionally more hip replacement admissions had less weekday LOS fluctuations. These findings suggest that hospitals which are “proxy”-specialized in managing hip replacement perform better in terms of LOS.

Our study compared hospitals of different ownership status (government, for-profit, and nonprofit), and found that they differ in terms of the LOS and for the ‘LOS Weekday Trend index’. Government hospitals keep hip replacement patients longer compared to for-profit (+0.167 days) and nonprofit (+0.267 days) ones. Differences were also observed between hospitals which are accredited and critical access ones. Non-accredited hospitals tend to keep hip replacement patient admissions for more days, on average. Similarly, the ‘LOS Weekday Trend Index’ differs based on a series of hospital attributes, such as the accreditation status of the hospital and the availability of emergency department.

When multivariate analysis was conducted to study the combined effect of the hospital structure and hospital process attributes on the LOS, we found a number of structure and process attributes to be associated with the hip replacement LOS. The hospital structure attributes ‘government hospital’ status and ‘critical access’ status were statistically significantly associated with an increased LOS, while the hospital process attribute ‘LOS Weekday Stability Index’ and the concentration of hip-replacement admissions on late weekdays (Thursday through Friday) were associated with increased LOS. On the other hand, the hip replacement admission ratio over total admissions was associated with a significantly lower LOS, indicating that hospitals with proportionally more hip replacement cases can manage these admissions more efficiently, resulting to shorter stays.

As healthcare costs continue to increase, hospitals should be aware of these findings as it pertains to finance and loss. Existing evidence agrees with our findings: Weekend admission is associated with an increase in cost and length of stay [19], with an increased length of stay contributing to the higher cost. In addition, weekend admission is associated with an increased hospital-acquired condition rate [20,21]. According to the Centers for Medicaid and Medicare Services, hospital-acquired conditions (HACs) are preventable adverse events that do not qualify for reimbursement of resulting hospitals costs [20]. Not only will hospitals not be reimbursed for patients who acquire hospital-acquired conditions, but revenue will continue to be lost due to beds staying occupied. In terms of DRGs, hospitals are paid a fixed amount based on the DRG or diagnosis, rather than paying the hospital what is spent caring for a hospitalized patient [22], including the LOS. This means, the longer a Medicare patient stays hospitalized, the more profit a hospital is losing.

Hospitals should also be aware that longer LOS leads to higher patient dissatisfaction [23]. Patient satisfaction is important, as it is commonly used for measuring quality in healthcare settings, particularly among doctors and hospitals [24]. To keep patients remaining loyal to a hospital, satisfaction is an important consideration. From the patient perspective, being admitted on the weekend should come with hesitancy. Research has shown that those admitted on the weekend are at higher risk for postoperative complications, as well as in-hospital mortality [25,26]. For a patient, a longer LOS may result in added stress due to the hospital environment, interrupted schedules, and the risk of developing a HAC. It is well known that increased stress leads to decreased wound healing [27], therefore prolonging the overall recovery time for a patient. Hospitals should also consider monitoring the clinical process and care transition electronically, as it has been suggested that this can have a positive effect on the LOS [28].

## 5. Conclusions

Our research adds important new knowledge on existing evidence as far as LOS performance management is concerned, for in-patient cases. Results furthermore provide evidence for associations, as far as the—very frequent—elective hip replacement hospital admissions are concerned. Hospital administrators can use these findings to make and communicate decisions to clinicians regarding optimal admission patterns to improve LOS performance. By keeping the LOS to a minimum, and in accordance with CMS standards, hospitals will see an increase to their revenue, an increase in patient satisfaction, and potentially improved in-patient and hospital-acquired infection and mortality rates. Towards this direction, we recommend to hospitals that they consider admitting hip replacement patients at the beginning of the week, without overconcentrating the admissions on just one day. We also recommend that hospitals should focus effort on maintaining a uniform care and case management pattern regardless of the weekday of admission for hip replacement surgeries and to account for structure and human resource related weekend slowdowns: Care transition and discharge decisions for hip replacement patients should not be made based on the (non)-availability of health professionals and should be universal and independent of weekday resource variations, which should, in turn, be eliminated to the extent possible.

## Figures and Tables

**Table 1:** Study attributes and description.

Attributes	Description	Comment
Inpatient Limited Medical Claims Dataset (2016)		
Admission Date	Date the beneficiary was admitted to the hospital, skilled nursing facility, or religious nonmedical health care institution.	Used to extract the admission weekday
Discharge Date	The date the beneficiary was discharged from the facility or died.	Combined with the admission date, for the LOS estimation
DRG Code	The diagnostic related group to which a hospital claim belongs for prospective payment purposes. It is determined by the ‘Grouper’ software.	The dataset was filtered to only keep cases with DRG = 470
Type of Admission	1 = Emergency, 2 = Urgent, 3 = Elective	The dataset was filtered to only include elective cases
Medicare Provider ID	The Medicare Provider Identification Number	
American Hospital Association Data Set (2016)		
Medicare Provider ID	Provider Identification Number	
Ownership Type	Government; For-Profit; Nonprofit	
Beds (total facility)	Total number of beds in the given facility	

**Table 2:** Descriptive statistics.

**Attribute Description**	**N**	**Mean**	**Std. Dev.**	**Min.**	**Max.**
‘LOS Weekday Trend Index’	2721	0.178	0.636	−1.000	1.000
‘LOS Weekday Stability Index’	2728	0.599	0.630	0.000	8.287
Mean LOS	2805	2.621	0.633	0.432	10.647
Admission Ratio of DRG = 470 over total	2805	0.079	0.123	0.001	0.909
Total Admissions	2805	313.34	3292.372	16	36686
Total Beds	2775	225.37	225.772	3	2877
Proportion of DRG = 470 admissions on Monday	2805	32.255	22.831	0.000	100
Proportion of DRG = 470 admissions on Tuesday	2805	32.328	22.912	0.000	100
Proportion of DRG = 470 admissions on Wednesday	2805	18.586	17.761	0.000	100
Proportion of DRG = 470 admissions on Thursday	2805	10.599	13.080	0.000	100
Proportion of DRG = 470 admissions on Friday	2805	5.907	9.310	0.000	96.667
Proportion of DRG = 470 admissions on Saturday	2805	0.193	0.896	0.000	20.000
Proportion of DRG = 470 admissions on Sunday	2805	0.128	0.817	0.000	22.641
Weekday DRG = 470 Admission Distribution	2805	21.474	9.554	2.213	44.721
**Attribute Description**	***N* (Valid Percent)**				
	**Yes**		**No**		
ACGME Accreditation	1298 (46.8%)		1477 (53.2%)		
American Osteopathic Association Residency	250 (9.0%)		2525 (91.0%)		
Critical Access Hospital	284 (10.2%)		2491 (89.8%)		
DNV Accreditation	266 (9.6%)		2509 (90.4%)		
Joint Commission Accreditation	600 (21.6%)		2175 (78.4%)		
Council of Teaching Hospitals AAMC	217 (7.8%)		2558 (91.1%)		
Ambulance Services	398 (17.7%)		1855 (82.3%)		
Assisted Living Services	66 (2.9%)		2187 (97.1%)		
Adult Cardiology Services	1777 (78.9%)		476 (21.1%)		
Emergency Department	2208 (98.0%)		45 (2.0%)		
Freestanding Outpatient Center	1128 (49.9%)		1125 (50.1%)		
Home Health Services	617 (27.4%)		1636 (72.6%)		
Hospital Based Outpatient Services	1997 (88.6%)		256 (11.4%)		
Mobile Health Services	389 (17.3%)		1864 (82.7%)		
>50% of DRG = 470 Admissions in just one weekday	1240 (44.2%)		1565 (55.8%)		
>90% of DRG = 470 Admissions in two weekdays	822 (29.3%)		1983 (70.7%)		
Ownership Status	Government: 343 (12.24%) For Profit: 528 (19.0%) Nonprofit: 1904 (68.6%)				

**Table 3:** Distribution of admissions per weekday (%): All hospitals.

Day	% of Admissions
Monday	30.48
Tuesday	30.80
Wednesday	19.40
Thursday	11.85
Friday	7.15
Saturday	0.19
Sunday	0.09

**Table 4:** Association between weekday DRG = 470 admission distribution and mean LOS.

Correlation Statistic	DRG = 470 Weekday Admission Distribution x LOS						
	Mon	Tue	Wed	Thu	Fri	Sat	Sun
Pearson	−0.055	0.012	−0.043	0.075	0.061	0.106	0.127
Sig. (2-tailed)	0.003	0.508	0.023	0.000	0.001	0.000	0.000

**Table 5:** Post-hoc comparisons between hospitals of different ownership status.

Dependent Variable	(I) Ownership	(J) Ownership	Mean Difference (I–J)	S.E	Sig.	95% CI
LOS Trend Index	1 Government	2 For Profit	0.142	0.045	0.005	0.036–0.248
		3 Nonprofit	0.029	0.038	0.719	−0.060–0.120
	2 For Profit	1 Government	−0.142	0.045	0.005	−0.248–−0.036
		3 Nonprofit	−0.112	0.031	0.001	−0.186–−0.038
	3 Nonprofit	1 Government	−0.029	0.038	0.719	−0.120–0.060
		2 For Profit	0.112	0.031	0.001	0.038–0.186
LOS Stability Index	1 Government	2 For Profit	0.040	0.044	0.630	−0.063–0.145
		3 Nonprofit	0.052	0.038	0.352	−0.036–0.141
	2 For Profit	1 Government	−0.040	0.044	0.630	−0.145–0.063
		3 Nonprofit	0.011	0.031	0.929	−0.061–0.084
	3 Nonprofit	1 Government	−0.052	0.038	0.352	−0.141–0.036
		2 For Profit	−0.011	0.031	0.929	−0.084–0.061
Mean LOS	1 Government	2 For Profit	0.167	0.043	0.000	0.065–0.269
		3 Nonprofit	0.267	0.036	0.000	0.181–0.354
	2 For Profit	1 Government	−0.167	0.043	0.000	−0.269–−0.065
		3 Nonprofit	0.100	0.030	0.003	0.028–0.173
	3 Nonprofit	1 Government	−0.267	0.036	0.000	−0.354–−0.181
		2 For Profit	−0.100	0.030	0.003	−0.173–−0.028

**Table 6:** Comparison of LOS between hospital characteristics and services availability.

Hospital Attribute	F	Sig.	*t*	df	Sig	Mean Diff	S.E Diff	95% C.I of Diff.
ACGME Accreditation	0.590	0.442	4.185	2773	0.000	0.100	0.024	0.053–0.147
Critical Access Hospital	0.174	0.677	−7.527	2773	0.000	−0.295	0.039	−0.372–−0.218
Joint Commission Accreditation	2.495	0.114	3.956	2773	0.000	0.115	0.029	0.058–0.172
Ambulance services	3.712	0.054	−2.498	2251	0.013	−0.087	0.034	−0.155–−0.018
Adult Cardiology Services	0.222	0.637	3.336	2251	0.001	0.108	0.032	0.044–0.172

**Table 7:** Comparison of ‘LOS Weekday Trend Index’ scores between hospital characteristics/services.

Hospital Attribute	F	Sig.	t	df	Sig	Mean Diff	S.E Diff.	95% C.I of Diff.
ACGME Accreditation	65.986	0.000	−4.059	2690	0.000	−0.099	0.024	−0.147–−0.051
American Osteopathic Assoc. Residency	23.267	0.000	−2.129	2690	0.033	−0.090	0.042	−0.173–−0.007
Critical Access Hospital	69.562	0.000	2.113	2690	0.035	0.090	0.042	0.006–0.174
Council of Teaching Hospitals AAMC	6.421	0.011	−2.284	2690	0.022	−0.103	0.045	−0.192–−0.014
Ambulance services	1.926	0.165	−2.194	2182	0.028	−0.077	0.035	−0.146–−0.008
Emergency Department	0.001	0.981	−3.081	2182	0.002	−0.297	0.096	−0.487–−0.108
Adult Cardiology Services	62.464	0.000	−2.577	2182	0.010	−0.085	0.033	−0.150–−0.020

**Table 8:** Comparison between LOS and DRG = 470 admission overconcentration.

Overconcentration Index	F (Sig.)	*t*	df	Sig.	Mean Diff	S.E. Diff.	95% CI of Diff.
>50% of admissions in a single weekday	0.006 (0.936)	−7.183	2803	0.000	−0.171	0.023	−0.218–−0.124
>90% of DRG = 470 admissions in two days	0.113 (0.736)	−5.696	2803	0.000	−0.148	0.026	−0.200–−0.097

**Table 9:** Multiple linear regression coefficients.

Independent Variable	Collinearity Statistics					
	B	S.E.	*t*	Sig.	Tolerance	VIF
(Constant)	3.191	0.140	22.724	0.000		
**Hospital Structure Attributes**						
Ownership = Government	0.168	0.041	4.098	0.000	0.783	1.277
Ownership = For Profit	0.318	0.042	7.502	0.000	0.686	1.458
Total Beds	0.000	0.000	−2.605	0.009	0.364	2.745
Joint Commission Accreditation	−0.077	0.041	−1.894	0.058	0.513	1.949
Council of Teaching Hospital AAMC	0.095	0.052	1.834	0.067	0.599	1.671
Assisted Living Services	0.002	0.077	0.023	0.982	0.902	1.109
Critical Access Hospital	0.200	0.052	3.883	0.000	0.635	1.576
Emergency Department	−0.302	0.099	−3.050	0.002	0.736	1.358
Freestanding Outpatient Center	0.018	0.028	0.643	0.520	0.703	1.422
Home Health Services	0.018	0.029	0.633	0.527	0.845	1.183
Hospital Based Outpatient Services	0.034	0.040	0.837	0.403	0.858	1.166
Mobile Health Services	0.031	0.035	0.893	0.372	0.791	1.265
Ambulance Services	0.033	0.034	0.956	0.339	0.812	1.232
Adult Cardiology Services	−0.083	0.039	−2.122	0.034	0.555	1.800
**Hospital Process Attributes**						
LOS Weekday Trend Index	−0.017	0.020	−0.880	0.379	0.909	1.101
LOS Weekday Stability Index	0.134	0.020	6.732	0.000	0.870	1.149
DRG = 470 Admission Ratio (over total admissions)	−1.926	0.148	−13.038	0.000	0.543	1.843
Proportion of DRG = 470 Admissions on Monday	−0.001	0.001	−1.876	0.061	0.720	1.389
Proportion of DRG = 470 Admissions on Wednesday	0.000	0.001	−0.250	0.802	0.779	1.283
Proportion of DRG = 470 Admissions on Thursday	0.005	0.001	4.457	0.000	0.745	1.343
Proportion of DRG = 470 Admissions on Friday	0.006	0.001	4.518	0.000	0.808	1.238
Proportion of DRG = 470 Admissions on Saturday	0.033	0.014	2.319	0.020	0.842	1.188
Proportion of DRG = 470 Admissions on Sunday	0.060	0.016	3.857	0.000	0.794	1.259
>50% of DRG = 470 admissions in one day	0.057	0.033	1.734	0.083	0.534	1.872
>90% of DRG = 470 admissions in two days	−0.023	0.037	−0.620	0.535	0.519	1.928

Dependent variable: Mean LOS of DRG = 470 admissions.

## References

[1] (2018). Health Care Use—Length of Hospital Stay—OECD Data. http://data.oecd.org/healthcare/length-of-hospital-stay.htm.

[2] Clarke A. (1996). Why are we trying to reduce length of stay? Evaluation of the costs and benefits of reducing time in hospital must start from the objectives that govern change. Qual. Health Care.

[3] Carey K. (2000). Hospital Cost Containment and Length of Stay: An Econometric Analysis. South. Econ. J..

[4] Earnest A., Chen M.I., Seow E. (2006). Exploring if day and time of admission is associated with average length of stay among inpatients from a tertiary hospital in Singapore: An analytic study based on routine admission data. BMC Health Serv. Res..

[5] Ryan K., Levit K., Davis P.H. (2010). Characteristics of Weekday and Weekend Hospital Admissions. https://www.ncbi.nlm.nih.gov/books/NBK53602/.

[6] Pauls L.A., Johnson-Paben R., McGready J., Murphy J.D., Pronovost P.J., Wu C.L. (2017). The Weekend Effect in Hospitalized Patients: A Meta-Analysis. J. Hosp. Med..

[7] Cram P., Hillis S., Barnett M., Rosenthal G.E. (2004). Effects of weekend admission and hospital teaching status on in-hospital mortality. Am. J. Med..

[8] Blecker S., Goldfeld K., Park N., Shine D., Austrian J.S., Braithwaite R.S., Radford M.J., Gourevitch M.N. (2014). Electronic health record utilization, intensity of hospital care, and patient outcomes. Am. J. Med..

[9] Gray B.H., (US) Institute of Medicine (1983). Physician Involvement in Hospital Decision Making.

[10] Medicine J.H. (2018). Types of Surgery|Johns Hopkins Medicine Health Library. https://www.hopkinsmedicine.org/healthlibrary/conditions/surgical_care/types_of_surgery_85,P01416.

[11] What Is Elective Surgery?. https://kidshealth.org/en/parents/elective.html.

[12] Aylin P., Alexandrescu R., Jen M.H., Mayer E.K., Bottle A. (2013). Day of week of procedure and 30 day mortality for elective surgery: Retrospective analysis of hospital episode statistics. BMJ.

[13] System L.U.H. (2018). Surgical Site Infections are the Most Common and Costly of Hospital Infections: Guidelines for Preventing Surgical Site Infections are Updated. https://www.sciencedaily.com/releases/2017/01/170119161551.htm.

[14] Maradit Kremers H., Larson D.R., Crowson C.S., Kremers W.K., Washington R.E., Steiner C.A., Jiranek W.A., Berry D.J. (2015). Prevalence of Total Hip and Knee Replacement in the United States. J. Bone Joint Surg. Am..

[15] The Medicare Beneficiary Population (2018). AARP Public Policy Institute. https://assets.aarp.org/rgcenter/health/fs149_medicare.pdf.

[16] NHE-Fact-Sheet (2018). Centers for Medicare and Medicaid Services. https://www.cms.gov/research-statistics-data-and-systems/statistics-trends-and-reports/nationalhealthexpenddata/nhe-fact-sheet.html.

[17] Diagnosis Related Group (DRG). https://hmsa.com/portal/provider/zav_pel.fh.DIA.650.htm.

[18] Bueno H., Ross J.S., Wang Y., Chen J., Vidán M.T., Normand S.L., Curtis J.P., Drye E.E., Lichtman J.H., Keenan P.S. (2018). Trends in Length of Stay and Short-term Outcomes Among Medicare Patients Hospitalized for Heart Failure, 1993–2006. JAMA.

[19] Attenello F.J., Wen T., Cen S.Y., Ng A., Kim-Tenser M., Sanossian N., Amar A.P., Mack W.J. (2015). Incidence of “never events” among weekend admissions versus weekday admissions to US hospitals: National analysis. BMJ.

[20] Wen T., Pease M., Attenello F.J., Tuchman A., Donoho D., Cen S., Mack W.J., Acosta F.L. (2015). Evaluation of Effect of Weekend Admission on the Prevalence of Hospital-Acquired Conditions in Patients Receiving Cervical Fusions. World Neurosurg..

[21] Attenello F.J., Wen T., Huang C., Cen S., Mack W.J., Acosta F.L. (2015). Evaluation of weekend admission on the prevalence of hospital-acquired conditions in patients receiving thoracolumbar fusions. J. Clin. Neurosci..

[22] Davis E. (2018). Learn About Diagnostic Related Grouping and How It Works. https://www.verywellhealth.com/drg-101-what-is-a-drg-how-does-it-work-3916755.

[23] Quintana J.M., González N., Bilbao A., Aizpuru F., Escobar A., Esteban C., San-Sebastián J.A., de-la-Sierra E., Thompson A. (2006). Predictors of patient satisfaction with hospital health care. BMC Health Serv. Res..

[24] Prakash B. (2010). Patient Satisfaction. J. Cutan Aesthet Surg..

[25] Zapf M.A., Kothari A.N., Markossian T., Gupta G.N., Blackwell R.H., Wai P.Y., Weber C.E., Driver J., Kuo P.C. (2015). The “weekend effect” in urgent general operative procedures. Surgery.

[26] An R. (2017). Impact of weekend admission on in-hospital mortality among U.S. adults, 2003–2013. Ann. Epidemiol..

[27] Christian L.M., Graham J.E., Padgett D.A., Glaser R., Kiecolt-Glaser J.K. (2018). Stress and Wound Healing. Neuroimmunomodulation.

[28] Zikos D., Diomidous M., Mpletsa V. (2014). The effect of an electronic documentation system on the trauma patient’s length of stay in an emergency department. J. Emerg. Nurs..

